# Chemopreventive Efficacy of Sulindac Sulfone as a Selective Apoptotic Antineoplastic Drug in Human Head and Neck Squamous Cell Carcinoma Cell Lines: A Systematic Review

**DOI:** 10.7759/cureus.51692

**Published:** 2024-01-05

**Authors:** Nivethitha Karuppiah, Sivapathasundharam B, Rajeswari M Chockalingam, Prem Karthick Bhupathy, Gnanambigai Kalaimani, Raghini Ramamurthi

**Affiliations:** 1 Oral Pathology and Microbiology, Priyadarshini Dental College and Hospital, Tiruvallur, IND; 2 Oral and Maxillofacial Pathology, Priyadarshini Dental College and Hospital, Thiruvallur, IND; 3 Oral Pathology, Priyadarshini Dental College and Hospital, Thiruvallur, IND; 4 Oral Pathology, Madha Dental College and Hospitals, Chennai, IND

**Keywords:** anti-neoplasm, cell lines, anti-inflammatory, sulindac sulfone, oral cancer

## Abstract

Sulindac sulfone, an active metabolite of sulindac, a non-steroidal anti-inflammatory drug, has good anti-inflammatory potential. The antineoplastic effect of sulindac sulfone is mediated through a cyclooxygenase inhibitory mechanism, followed by apoptosis and inhibition of cell proliferation. Mounting studies have explored the anti-neoplastic effect of sulindac sulfone in various types of cancers in a dose-dependent manner. In this backdrop, we have conducted a systematic review to evaluate the efficacy and dose of sulindac sulfone as an anti-neoplastic agent in human head and neck squamous cell carcinoma cell lines (HNSCCs). In this study, we used a systematic literature review approach, and articles were searched in PubMed, and Medline with the keywords “sulindac sulfone,” “anti-neoplastic activity,” “chemopreventive,” and “head and neck squamous cell carcinoma”. A hand-search of journals was also performed. Articles were reviewed and analyzed. The analysis reveals that, based on the in vitro studies on various tumor models, the optimum concentration of sulindac sulfone which elicits anti-neoplastic effects is 200-800 µM. The anti-neoplastic effect is mediated through inhibition of cell proliferation and apoptosis. The results of our systematic review show that the anti-neoplastic activity of pharmacologic Sulindac sulfone is part of its dose-dependent activity, which can be safely employed in the therapy for human HNSCCs and would be responsible for a beneficial outcome of the treatment.

## Introduction and background

Squamous cell carcinoma of the head and neck is the sixth most common cancer globally and accounts for 3% of all cancer types [1, 2]. Nearly 310,000 new cases of oral and pharyngeal cancers are reported annually, and they account for a significant cause of mortality and morbidity [3]. Lifestyle modifications such as avoiding tobacco use (chewing and smoking), betel nut, and alcohol consumption are advised, but these preventive measures are not always effective [4]. The current treatment strategies for head and neck squamous cell carcinoma cell lines (HNSCCs) include multimodal therapy, which encompasses chemoprevention, surgery, chemoradiation, or a combination thereof [5].

Sulindac sulfone is a chemotherapeutic agent used in managing a wide range of cancers, including those of the colon, prostate, and lung [6, 7, 8]. However, its efficacy in managing human HNSCCs is still unclear. Sulindac sulfone is a biologically active metabolite of sulindac, a non-steroidal anti-inflammatory drug that belongs to the class of indole derivatives. The half-life of sulindac is approximately six hours, and its efficacy is maximized when consumed orally. The metabolism of sulindac yields two major metabolites: sulindac sulfone and sulfide.

The anti-neoplastic effect of sulindac sulfone is mediated through three cardinal mechanisms: cell growth inhibition, decreased cell proliferation, and induction of apoptosis [9, 10]. Additionally, the anti-inflammatory activity of sulindac sulfone is mediated through a COX inhibition mechanism and through the inhibition of various inflammatory cytokines. Thus, these effects play a significant role in the anti-cancer effect of sulindac sulfone, promoting it as a novel anti-neoplastic drug in the therapeutic management of HNSCC [11, 12].

The cell proliferation and cell growth inhibition by sulindac sulfone are mediated through a COX inhibitory-dependent mechanism [13]. PPARs (peroxisome proliferator-activated receptors) are nuclear hormone receptors that serve as ligand-activated transcription factors for cell proliferation and growth. Additionally, during COX activity, some prostaglandins are released and function as ligands for PPARs. Sulindac, during COX inhibition, also blocks the release of prostaglandins, thus hindering PPAR activation and blocking the transcriptional activity of cell proliferation and growth by PPARs [14]. Meanwhile, sulindac sulfone-mediated apoptosis is rendered through a COX-independent mechanism and involves a decrease in cellular levels of cyclic GMP phosphodiesterase enzyme, leading to increased levels of cyclic GMP, which further activates protein kinase G (PKG). This activation leads to the phosphorylation of β-catenin, which in turn leads to proteasome degradation [15, 16]. The degradation of β-catenin further triggers apoptosis through various pathways [16].

Mounting in-vitro studies have reported the anti-cancer effect of sulindac in HNSCCs. These cell lines represent clones of immortal cells derived from HNSCC patients and exhibit a uniform genetic arrangement. Cell lines derived from human cells serve as an important model for evaluating novel drugs and new chemical entities for managing head and neck cancer [17]. Meanwhile, in vitro evaluation of drugs offers many merits, such as the homogeneity of samples used, reduced cost, and avoidance of legal issues associated with animal use.

With credible clinical and biological evidence for the chemotherapeutic effect of sulindac sulfone reported, we conducted a systematic review of studies delineating the chemotherapeutic effect of sulindac sulfone in human HNSCCs.

## Review

Methods

Search Strategy for Identification of Studies

The present systematic review was conducted according to the guidelines of the Preferred Reporting Items for Systematic Reviews and Meta-Analyses (PRISMA) statement. The search strategy adhered to the Cochrane guidelines for systematic reviews. Articles were searched and selected using PubMed and Medline up to the year 2023. Due to the scarcity of studies conducted on the tissues of the oral cavity, we aimed to exhaust all possible articles; therefore, a timeline was not included in the search. The article search was limited to those published in English. An internet search was also conducted to obtain relevant articles of interest. The titles and abstracts of the articles were reviewed.

Search Methodology

The search through PubMed was conducted using the following keywords: (sulindac sulfone) OR (exisulind) OR (1H-indene 3-acetic acid 5-fluoro-2-methyl) OR (FGN-1) OR (aptosyn) AND (chemopreventive) OR (chemotherapy) OR (antineoplastic drug) OR (anticancer agent) OR (cancer chemotherapy) AND (squamous cell carcinoma) OR (oral cancer) OR (head and neck squamous cell carcinoma) OR (squamous cell carcinoma of the head and neck) AND (cell cultures) OR (in vitro) OR (cell lines).

**Figure 1:**
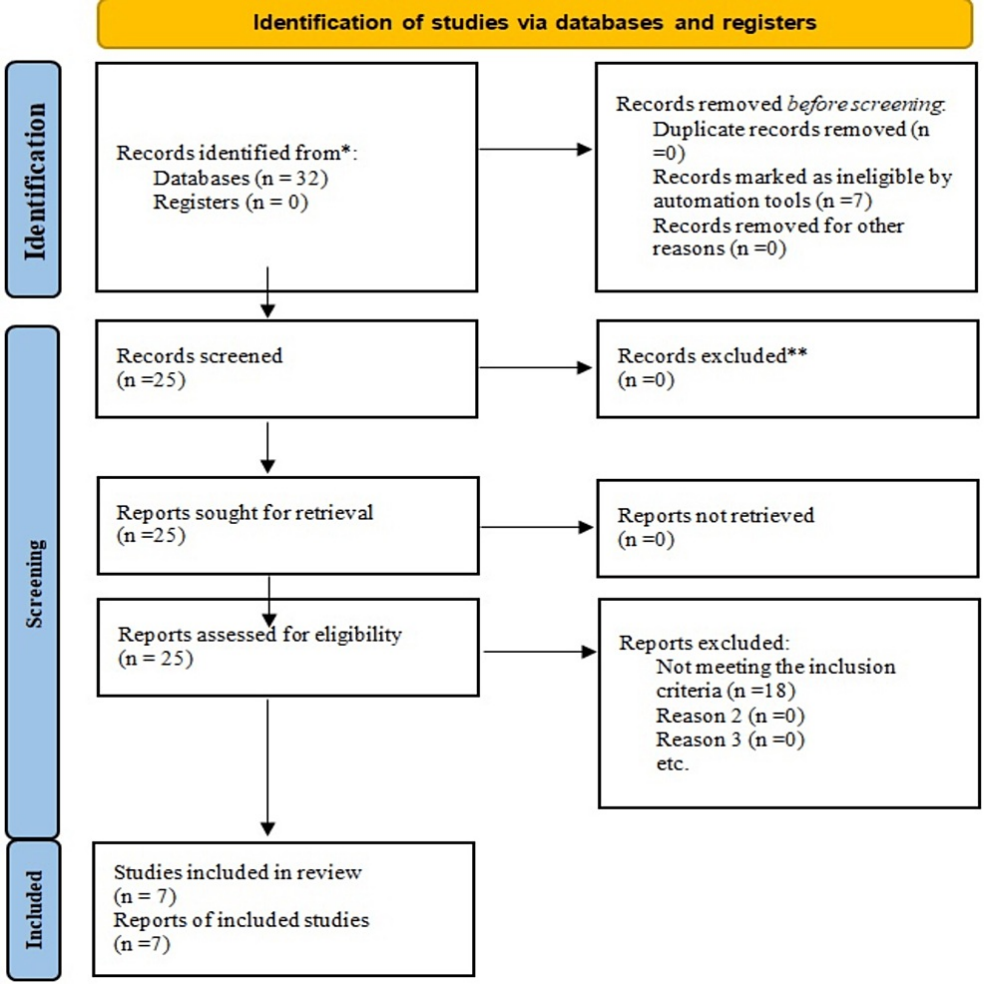
PRISMA flow diagram illustrating the search strategy and study selection process for the systematic review.

In addition, an internet search was conducted using the keywords "sulindac sulfone," "antineoplastic activity," "head & neck squamous cell carcinoma," and "cell lines." Journals evaluating the antineoplastic role of sulindac sulfone in human head & neck squamous cell carcinoma were also consulted from cross-references. The process was summarized in a search flowchart (Figure 1).

Inclusion Criteria

Inclusion criteria for the systematic review encompass studies that evaluated the pharmacologic dose-associated antineoplastic activity of sulindac sulfone, exisulind, or FGN-1 in human HNSCCs; those that analyzed the optimum dose effects of sulindac sulfone, which also include esophageal, laryngeal, and thyroid carcinoma; studies employing sulindac sulfone along with sulindac sulfide; research evaluating the effects of sulindac sulfone in conjunction with other NSAIDs on human HNSCCs; and studies assessing the antineoplastic effects of sulindac sulfone and its derivative (CP248) in the specified cell lines.

Exclusion Criteria

Studies that have investigated the effects of sulindac sulfone in patients with colon carcinoma, prostate carcinoma, lung carcinoma, urinary bladder cancer, gastric carcinoma, thyroid carcinoma, thyroid carcinoma cell lines along with thyroid carcinoma patients, laryngeal squamous cell carcinoma cell lines along with nude mice, animal models, human colon carcinoma cell lines, and review articles were excluded from the study.

Data Extraction and Analysis

Once the potentially relevant articles for the systematic review were obtained, the data extracted from each article were tabulated and later cross-checked.

Outcomes

The outcomes assessed in this review were the optimum concentration or level of sulindac sulfone required to exert an antineoplastic effect in vitro, the effects of pharmacologic dose supplementation of sulindac sulfone on human HNSCC, and the mechanism of action exhibited by sulindac sulfone which brings anticancer activity to human HNSCC.

Results****

The search strategy identified seven studies that evaluated the anti-neoplastic activity of sulindac sulfone in various human HNSCC cell lines. The search strategy included all in vitro studies. The parameters measured varied from study to study when evaluating the anti-neoplastic effects of sulindac sulfone on human HNSCC. Most studies measured the percentages and mean & standard deviations of apoptotic activity, antiproliferative activity, cell cycle progression, inhibition of the G1 cell cycle phase, and β-catenin and E-cadherin levels in various human HNSCC cell lines. The description of the included studies is shown in Table 1.

**Table 1:** Description of studies of the selected papers for analysis E, enzyme-linked immunosorbent assay; FC, flow cytometry; IHC, immunohistochemistry; IMF, immunofluorescence; WB, western blot; HPLC, high-performance liquid chromatography; PCR, polymerase chain reaction.

S. No.	Reference	Type of Study	Agent Tested	Co-administered Agents	Cell Type	Concentration of Sulindac Sulfone										Method of Assessment							
						10 µM	50 µM	100 µM	150 µM	200 µM	300 µM	400 µM	500 µM	600 µM	800 µM	E	FC	IHC	IMF	WB	HPLC	PCR	
1	Bock et al. 2007 [13]	In vitro study	Sulindac sulfone	Sulindac sulfide	UMSCC-1 and 25 cell lines	+	+	+	–	+	–		–			+	+	–	–	–	–	–	
2	Nikitakis et al. 2002 [14]	In vitro study	Sulindac sulfone	Sulindac sulfide	Human oral squamous cell carcinoma cell lines. (SCC-4, 9, 15, and 25)	–	+	+	+	–	–	–	+	–	–	–	+	–	–	–	–	+	
3	Sauter et al. 2010 [16]	In vitro study	Sulindac sulfone (Ss)		University of Michigan squamous cell carcinoma cell lines (UMSCC-11 A) cell lines	–	–	+	–	+	–	+	–	+	+	+	–	+	–	–	–	–	
																							4	Bock et al. 2007 [18]	In vitro study	Sulindac sulfone	Celecoxib, rofecoxib, indomethacin, ketoprofen flurbiprofen naproxen, piroxicam, aspirin	UMSCC-1 cell lines	–	–	+	–	+	+	+	+	–	–	+	+	–	–	–	–	–	
																							5	Naim et al. 2006 [19]	In vitro study	Sulindac sulfone		UMSCC cell lines	–	–	+	–	+	–	+	–	+	+	+	–	–	–	–	–	–	
																							6	Joe et al. 2003 [20]	In vitro study	Exisulind (sulindac sulfone)	CP248, a derivative of sulindac sulfone	Barrett’s esophagus-associated adenocarcinoma cell lines Seg-1 and Bic-1. Esophageal squamous cell carcinoma cell lines.	–	+	+	–	+	+	+	+	–	–	–	+	–	+	+	+	–	
																							7	Quidville et al. 2006 [21]	In vitro study	Sulindac sulfone	Ibuprofen, sulindac sulfide, aspirin, celecoxib, NS398, SC560	Human medullary thyroid carcinoma cell lines obtained from the American type culture collection	–	+	+	–	–	–	–	–	–	–	–	+	–	–	–	–	–	

The optimum concentration of sulindac sulfone used to produce a potential anti-neoplastic effect was found to be 200µM, 400µM, and 800µM in most of the HNSCC cell lines. These pharmacologic concentrations were found to be effective against tumor cells, mediated by various mechanisms such as the induction of decreased cell proliferation, cell growth inhibition, and apoptosis. Thus, we infer that the pharmacologic doses of sulindac sulfone can be safely employed in the treatment of human HNSCC. The data are shown in Table 2.

**Table 2:** Outcome of the studies included in the analysis MTT: dimethylthiazol-diphenyltetrazolium bromide; JNK: c-Jun N-terminal kinases; COX-2: cyclooxygenase-2; GSH: reduced glutathione; PPARα- peroxisome proliferator-activated receptor-α.

Author and Year of Publication	Evaluated Parameters	Results
Bock et al. 2007 [13]	Cell growth inhibition – MTT assay (ELISA method)	At 200 µM – significant cell growth inhibition
	Apoptosis – Active caspase-3 assay (Flow cytometry	1. Positive staining for caspase: at 200 µΜ =2% . 2. Morphological changes and nuclear condensation at 200 µΜ =2%
	G1 cell cycle phase inhibition – BrdUrd pulse flow cytometric assay	1. Accumulation of cells in G1 phase: At 100 µMol: 31.3±1.7; at 200 µMol: 33.9±2.4. 2. Depletion of cells in S phase: At 100 µMol: 48.4±2.4; at 200 µMol: 46.7±3.5 3. Accumulation of cells in G2 +M phase: At 100 µMol: 24.1±4.9; at 200 µMol: 22.5± 4.4
Nikitakis et al. 2002 [14]	Cell growth inhibition – flow cytometry	At 50, 100, 150, 500 µMol sulindac sulfone in all four cell lines in normal medium: Significant main effect for time (F2,6 = 7.7, p≤0.05) and dosage (F3,9 = 5.8, p≤0.01)
	Apoptosis – (diploid peak of DNA) flow cytometry	At 50, 100, 150, 500 µMol sulindac sulfone in all four cell lines in fatty acid-free medium: Significant main effect for dosage (F3,9= 9.7, p ≤ 0.01)
	mRNA and protein	At 50, 100, 150, 500 µMol sulindac sulfone in all four cell lines: statistically significant increase in mRNA and protein expression (p ≤ 0.05)
	Expression of PPARs and COX-2 – quantitative reverse transcriptase – polymerized chain reaction	After 500 µMol sulindac sulfone: PPARα= 7.17, PPARβ/δ =1.94, PPARγ = 7.73, COX-2 = 1.48
Sauter et al. 2010 [16]	Percentage of β-catenin level (ELISA method)	At 800 µMol – 73% cells
	Percentage grading of immunostaining of β-catenin(immunohistochemistry)	1. At 100 µMol – 31% cells (strong reactivity) 2. At 200 µMol – 0% cells (strong reactivity) 3. At 400 µMol – 0 % cells (strong reactivity) 4. At 600 µMol – 0 % cells (strong reactivity) 5. At 800 µMol – 0% cells (strong reactivity)
Bock et al. 2007 [18]	Anti-proliferative activity – MTT assay	IC50 values: 315.8±1.1 µM
	Apoptotic activity – active caspase-3 assay	Caspase-3 activity: Minimal activity
	Inhibition of G1 cell cycle phase – BrdUrd flow cytometric assay	No significant G1 cell cycle phase inhibition
	Induction of p21 – immunoblotting	No significant induction of p21
	Inhibition of E2F transcription factor activity – Luciferase assay	No significant inhibition of E2F
Naim et al. 2006 [19]	Percentage of E-cadherin levels – ELISA method	1. At 100 µMol sulindac sulfone – 13.11%. 2. At 200 µMol sulindac sulfone – 16.41%. 3. At 400 µMol sulindac sulfone – 21.17%. 4. At 600 µMol sulindac sulfone – 29.51%. 5. At 800 µMol sulindac sulfone – 32.22%
Joe et al. 2003 [20]	Growth inhibition	Exisulind at 150–300 µM (48 hours) in all cell lines: statistically significant growth inhibition
	Percentage of apoptosis – annexin V-phycoerythrin (PE)-based immunofluorescence assay	At 300 µMol (aft 48 hours): 18.6 % in Bic-1 cells, (after 48 hours): 6.3% in Seg-1 cells
	Cell cycle progression – DNA flow cytometry	1. Exisulind in Bic-1 cells. 2. Exisulind in Seg-1 cells at G1 phase: 56.8±3.0; at G1 phase: 57.2±3.6; at S phase: 21.8±2.4; at S phase: 19.0±4.5; at G2/M phase: 21.3±1.1; and at G2/M phase: 23.7±1.0
	JNK1 activation – JNK1 kinase assays	Exisulind at 300 µM (after 2 and 24 hours): In Seg-1 cells: rapid activation of JNK1
	COX-2 induction – western blotting	Exisulind at 200 µM (after 48 hours): In Seg-1cells: expressed basal level of COX-2; in Bic-1 cells: not expressed basal level of COX-2; and in HCE7 cells: not expressed basal level of COX-2
	Induction of reduced glutathione – Perkin-Elmer high-performance liquid chromatography	Exisulind at 300 µM (after 3 and 24 hours). In Seg-1 cells: twofold increase in levels of GSH at 3 hours, six- to eightfold increase in levels of GSH at 24 hours. In Bic-1 cells: twofold increase in levels of GSH at 3 hours, six- to eightfold increase in levels of GSH at 24 hours. In HCE7 cells: twofold increase in levels of GSH at 3 hours, six- to eightfold increase in levels of GSH at 24 hours
Quidville et al. 2006 [21]	Percentage of cell proliferation	At 100 µMol (at day 6) – 40%
	Percentage of 15-hydroxyprostaglandindehydrogease activity – tritium release assay	At 100 µMol (from 2 to 6 days) – 50%

Discussion 

Squamous cell carcinoma of the head and neck (SCCHN) is a type of epithelial malignancy characterized by invasion, metastasis, high recurrence risk, and significant morbidity and mortality, severely hampering the quality of life. The treatment outcomes for standard management strategies, including surgery, radiation therapy, and chemotherapy, have not significantly improved over the past 2-3 decades. NSAIDs have been employed in managing familial colon cancer syndromes for the past two decades and also showed good efficacy against SCCHN in the early 1980s [11]. Based on credible scientific reports, we conducted a systematic review of the chemotherapeutic effect of sulindac sulfone in human HNSSC cell lines.

Sulindac sulfone, an irreversible active metabolite of NSAID, mediates its anti-inflammatory effect by inhibiting COX and blocking the synthesis of prostaglandins by interfering with arachidonic acid metabolism. The sulindac metabolites, sulindac sulfide, and sulindac sulfone, mediate their anti-neoplastic effects through both COX inhibitory dependent and independent mechanisms [11, 12]. The anti-cancer effect of sulindac sulfone is mediated by three important mechanisms: inhibition of cell growth, reduced cell proliferation, and apoptosis. The cell growth inhibition of sulindac sulfone is a COX inhibitory independent action, which starts by arresting the G1 cell cycle phase, upregulating p21, inhibiting cyclin D, and blocking the cell cycle progression from G1 to S phase [18]. Additionally, the COX inhibitory-dependent decreased cell proliferation effect of sulindac sulfone is mediated by inhibiting prostaglandins, which serve as a ligand for PPARs activity and thus their inhibition leads to decreased transcriptional activity of PPARs in cell proliferation. The apoptotic activity of sulindac sulfone is mediated by the downregulation of β-catenin levels through the activation of protein kinase G (PKG). Furthermore, the depleted β-catenin levels trigger the activation of mitogen-activated protein kinases (MAPK) and stress-activated kinase (SEK), which are the upstream activators of the c-JUN kinase pathway, the terminal effects of which further activate caspase-3 and PARP cleavage, ultimately causing apoptosis.

This systematic review delineates the mechanism of sulindac sulfone and reports it systematically. In this analysis, seven studies were included, with sulindac sulfone concentrations ranging from 10 to 800 µM. The predominant anticancer activity, around 71%, was achieved in concentrations ranging from 200 to 800 µM on HNSCC cell lines. The main mechanisms are apoptosis and cell growth inhibition, evaluated by molecular methods such as ELISA, immunohistochemistry, active caspase-3 activity, flow cytometry, MTT assay, and annexin V-phycoerythrin (PE)-based immunofluorescence assay [13,14,16,18-20]. Among these techniques, ELISA and flow cytometry were commonly used for measuring β-catenin levels and G1 cell cycle phase arrest. The studies revealed that Sulindac sulfone at concentrations of 200-800 µM significantly downregulated β-catenin levels and upregulated p21. Thus, β-catenin inhibition leads to the activation of the C-Jun kinase pathway and apoptosis. Meanwhile, the upregulation of p21 inhibits the progression of the cell cycle from the G1 phase to the S phase. However, the inhibition of cell proliferation accounts for a minor amount, which was assessed by quantitative reverse transcriptase polymerase chain reaction that measures the mRNA and protein expression of PPARs, where expression of PPARs was found to be significantly low. Among the various cell lines used in the included studies, UMSCC cell lines were more sensitive to treatment with sulindac sulfone and showed a significant effect upon administration. Meanwhile, studies analyzing the effect of Sulindac sulfone at concentrations <200 µM in HNSCC cell lines showed only minimal anti-neoplastic effects in methods such as MTT assay and active caspase-3 assay and were found to be less significant.

Limitations

There is a presence of publication bias in this review. The concentrations employed, time duration, parameters, and the units of the results for evaluation in all the studies are not homogeneous; therefore, the review proceeded as a heterogeneous study. If the method and modes of evaluating the anti-neoplastic activity could be more standardized with minimal data set, it would help by providing homogeneous data for systematic review in the future.

## Conclusions

From the results of our systematic review, we can conclude that the anti-cancerous role played by sulindac sulfone at the concentration of 200-800 µM is mediated through apoptotic activity. So this enables the implication of this NSAID in the chemotherapeutic management of HNSCCs with potential benefits.
